# Friction in soft biological systems and surface self-organization: the role of viscoelasticity

**DOI:** 10.1007/s12551-024-01248-9

**Published:** 2024-10-28

**Authors:** Ivana Pajic-Lijakovic, Milan Milivojevic, Peter V. E. McClintock

**Affiliations:** 1 Department of Chemical Engineering, Faculty of Technology and Metallurgy, University of Belgrade, Belgrade, Serbia; 2 Department of Physics, Lancaster University, Lancaster, LA1 4YB UK

**Keywords:** Friction, Viscoelasticity, Collective cell migration, Mechanical stress, Cell response, Inertial effects

## Abstract

Friction is a critical factor in the proper functioning of human organs as well as in the potential development of disease. It is also important for the design of diagnostic and interventional medical devices. Nanoscale surface roughness, viscoelastic or plastic deformations, wear, and lubrication all influence the functions of individual cells. The effects of friction in soft matter systems are quantified using different types of frictional coefficients, including the dynamic friction coefficient, friction-skin drag, and pressure drag. These coefficients are determined by the viscoelastic properties of the two systems in contact and their relative velocity. In this review, several biological systems are considered, including (i) epithelial tissues in contact with soft hydrogel-like implants, (ii) the collective migration of epithelial monolayers on substrate matrices, (iii) blood flow through blood vessels, and (iv) the movement of cancer cells past epithelial clusters along with the migration of epithelial cells within the cluster.

## Introduction

The concept of friction, the force that resists the relative movement of interacting surfaces, has been a longstanding focus of attention in science, technology, and biomedicine. Wear, the process of material loss through contact and movement, and lubrication are intimately connected to friction (Butt and Kappl 2018). Despite its historical and continuing importance, a comprehensive macroscopic theory of friction is still lacking, probably on account of the complexity of the subject. This impedes our ability to predict frictional forces between bodies. Friction predominantly arises at a concealed interface that is analytically challenging and hard to access experimentally. The presence of nanoscale surface roughness, viscoelastic or plastic deformation, wear, and lubrication exert strong but distinct influences on frictional behavior, making it difficult to distinguish and control their individual contributions in most situations (Butt and Kappl 2018).

Friction effects have been considered along the contact interface areas between (i) two solids, (ii) a solid and a fluid, and (iii) two fluids. The different forms of friction that have been formulated include (i) static, dry (Coulomb) friction, (ii) dynamic friction, (iii) rolling friction, (iv) pressure drag, (v) friction-skin drag, and (vi) wave-making drag. A contact interface area is defined as the boundary between two spatial regions occupied by different materials undergoing surface interaction (Butt and Kappl 2018). We start by discussing frictional effects between two solid systems. Static friction must be overcome to initiate movement between two impermeable solid bodies that are initially at rest. This particular form of friction has not been detected in biological systems. Cells possess the ability to react to shear stress at the biointerface by altering their morphology and dimensions, aligning themselves so as to reduce shear stress, and initiating collective cell migration through diverse cellular signaling pathways.

Dynamic friction between two impermeable surfaces accounts for the effects of lubrication and can be considered within three regimes depending on the thickness of a lubricating fluid, i.e., the Stribeck diagram: boundary lubrication (i.e., dry friction), mixed lubrication for thin films of lubricant, and hydrodynamic lubrication for thicker film of lubricant (Al-Bender et al. 2005; Butt and Kappl 2018). In this case, the dynamic friction coefficient can be estimated as (i) ${C}_{F}>0.1$ boundary regime for neglected lubrication; (ii) $0.01<{C}_{F}<0.1$ for mixed regime, which can include stick–slip effects; and (iii) $0.001<{C}_{F}<0.01$ for hydrodynamic regime for pronounced lubrication. While the boundary and mixed lubrication regimes account for the surface wear, which depends on the viscoelasticity/plasticity of the contact interface area, hydrodynamic lubrication depends primarily on the viscosity of the lubricant fluid and the shear rate along the interface. The dynamic friction coefficient ${C}_{F}$ is space–time dependent. Static friction is higher than dynamic friction and can be additionally reduced by the presence of a lubricant. Rolling friction is lower than dynamic friction. However, the mechanism of lubrication between deformable, permeable, and aqueous surfaces such as polymer hydrogels and multicellular surfaces is much more complex and cannot be simplified in the form of Stribeck regimes (Chau et al. 2020). In the context of aqueous hydrogels, the dynamic friction coefficient is influenced by the mesh size, indicating a complex fluid flow that occurs along the surfaces and from the bulk region of the hydrogel to the surface via its porous structure (Chau et al. 2020). This fluid flow has a feedback impact on the viscoelasticity of hydrogels along the interface.

In further consideration, it is necessary to discuss frictional effects caused by flow of Newtonian fluids in different geometries. Pressure drag, accompanied by viscous drag, induces frictional effects caused by Newtonian fluid flow past particle/droplet, while friction-skin drag accounts for frictional effects caused by fluid flow along the solid surface (i.e., wall effects). Resistance effects caused by oscillatory movement of a solid through a fluid induce wave-making drag. As already mentioned, the friction-skin drag coefficient similarly, as previously mentioned dynamic (stick–slip) friction coefficient, depends on slip effects along the interface. In the case of the friction-skin drag coefficient, sliding effects have been expressed in the form of the Navier slip equation (Ferrás et al. 2017). More intensive sliding, quantified by higher slip velocity, is capable of reducing the frictional-skin drag coefficient (Jimenez Bolanos and Vernescu 2017). The dimensionless pressure drag coefficient is higher than the friction-skin drag coefficient. However, the friction-skin drag coefficient becomes important during fluid flow through a porous environment, such as hydrogels, due to the large internal interface area. The corresponding dimensionless drag coefficients can be estimated by measurement of the dynamic pressure drop caused by fluid flow. These coefficients depend on the rheological behavior of the fluid, the fluid density, and the flow geometry such as the size and shape of the solid body for the case of pressure drag, the average diameter and volume fraction of pores within a hydrogel, and the surface roughness in the case of friction-skin drag. Due to complexity of the flow pattern, dimensionless drag coefficients have been expressed as functions of the Reynolds number in the form of empirical correlations for laminar, transient, and turbulent flows. The Reynolds number is equal to ${R}_{e}=\frac{{\rho }_{l}UL}{{\upeta }_{l}}$ (where ${\upeta }_{l}$ is the viscosity of fluid, ${\rho }_{l}$ is its density, $L$ is a characteristic length, $U=\frac{Q}{S}$ is the average velocity, $Q$ is the volumetric flow rate, and $S$ is the cross-sectional area).

Our discussion above refers to frictional effects between solids and between solids and Newtonian fluids. Many questions arise. How does viscoelasticity influence frictional effects? In the context of viscoelasticity, there is no sharp border between solid-like and liquid-like systems. We can discuss the diverse behavior of viscoelastic liquids and viscoelastic solids (Pajic-Lijakovic 2021). Viscoelastic systems that have been considered in the context of frictional effects include polymer-water or polymer-oil solutions, hydrogels, and biological tissues (Langille et al. 1989; Varshney and Steinberg 2018; Pitenis and Sawyer 2020; Faroughi and Del Giudice 2022; Vazquez et al. 2022; Clark et al. 2022; Pajic-Lijakovic et al. 2024b). Although dynamic friction has been extensively studied for a variety of systems, it is not clear whether frictional effects between viscoelastic solids can be considered as either dynamical friction or frictional-skin drag. The dimensionless dynamic friction coefficient correlates shear stress with normal stress along the interface, while the frictional-skin drag coefficient correlates fluid shear stress with inertial stress (i.e., dynamic pressure). Migrating epithelial monolayers on substrate matrices establish strong cell–cell adhesion contacts and behave as viscoelastic solids. These systems have been considered in the context of dynamical friction (Vazquez et al. 2022). Dynamical friction has been related to the remodeling of cell–matrix adhesion contacts (Vazquez et al. 2022). Oscillatory changes of cell velocity, and the mechanical stress accumulated within migrating epithelial collectives, pointed to long-time inertial effects (Serra-Picamal et al. 2012; Pajic-Lijakovic et al. 2024a). However, inertial stress is much lower than cell shear stress. In humans, normal physiological blood flow induces shear stresses of $1-5 \text{Pa}$ (Baeyens et al. 2016). The stress is highly oscillatory in arteries, of similar magnitude but less oscillatory in capillaries, and about tenfold less with minimal oscillations in veins (Paszkowiak and Dardik 2003). This blood shear stress of around 2–10 Pa induces frictional-skin drag, which can deform endothelial cells, while higher flow shear stress in the range of 10–20 Pa can disrupt HEK293 and C2C12 cells (Rahman et al. 2018).

In the case of viscoelastic fluids, it is necessary to take account of another dimensionless number, in addition to the Reynolds number, for characterization of flow-induced drag. For polymer solutions, it is the Weissenberg number expressed as ${W}_{i}={\tau }_{R}\frac{U}{L}$ (where ${\tau }_{R}$ is the stress relaxation time and $\frac{U}{L}$ is the average shear strain rate). Polymer solutions satisfy the condition that ${W}_{i}\ge 1$ (Groisman and Steinberg 1998). Multicellular systems are more complex than other soft matter systems. They are capable of self-organization via collective cell migration, driven by the interplay between biological mechanisms such as cell signaling and gene expression, while physical mechanisms are associated with tissue surface characteristics and viscoelasticity. The viscoelasticity caused by collective cell migration occurs on two time-scales. A time-scale of minutes corresponds to the relaxation of cell mechanical stress, while a time-scale of hours corresponds to the accumulation of residual stress, changes in cell velocity, and the corresponding strain. While epithelial systems have been treated as viscoelastic solids, cancer mesenchymal-like multicellular systems have been treated as viscoelastic liquids (Pajic-Lijakovic et al. 2024b). Mesenchymal cells establish weak cell–cell adhesion contacts and migrate as streams. Cell stress relaxes within many short-time stress relaxation cycles under constant: (i) strain per cycle for viscoelastic solids or (ii) strain rate per cycle for viscoelastic liquids (Pajic-Lijakovic et al. 2024b).

Frictional effects along the biointerfaces between (i) migrating epithelial monolayers and substrate matrix, (ii) endothelial cells in contact with blood flow, and (iii) epithelium and cancer in direct contact can reduce cell migration. They can even cause tissue inflammation accompanied by cell damage and, on that basis, affect tissue self-organization in a plethora of morphogenetic contexts such as embryo development, wound healing, and cancer. A central goal of regenerative medicine is to improve the biocompatibility of biomedical implants in order to reduce frictional stress between them and soft tissues (Bijukumar et al. 2018).

The main focus of the present review lies in pointing out the role of viscoelasticity in the generation of frictional effects along various types of interfaces. We will consider interfaces between two solids, two fluids, and between fluid and solid by applying a variety of theoretical approaches, using experimental data from the literature. After extracting the main characteristics of frictional stress in a variety of soft matter systems, we will discuss the cumulative effects of cell friction in biological systems and their impact on tissue self-organization. The biological systems and processes to be considered are (i) collective migration of epithelial monolayers on substrate matrices, (ii) collective migration of mesenchymal-like cancer cells past epithelial cluster within co-cultured spheroids, (iii) flow of blood along blood vessels, and (iv) friction along a non-migrating epithelium-implant biointerface.

## Frictional effects in non-biological systems: the role of viscoelasticity

**Fig. 1:**
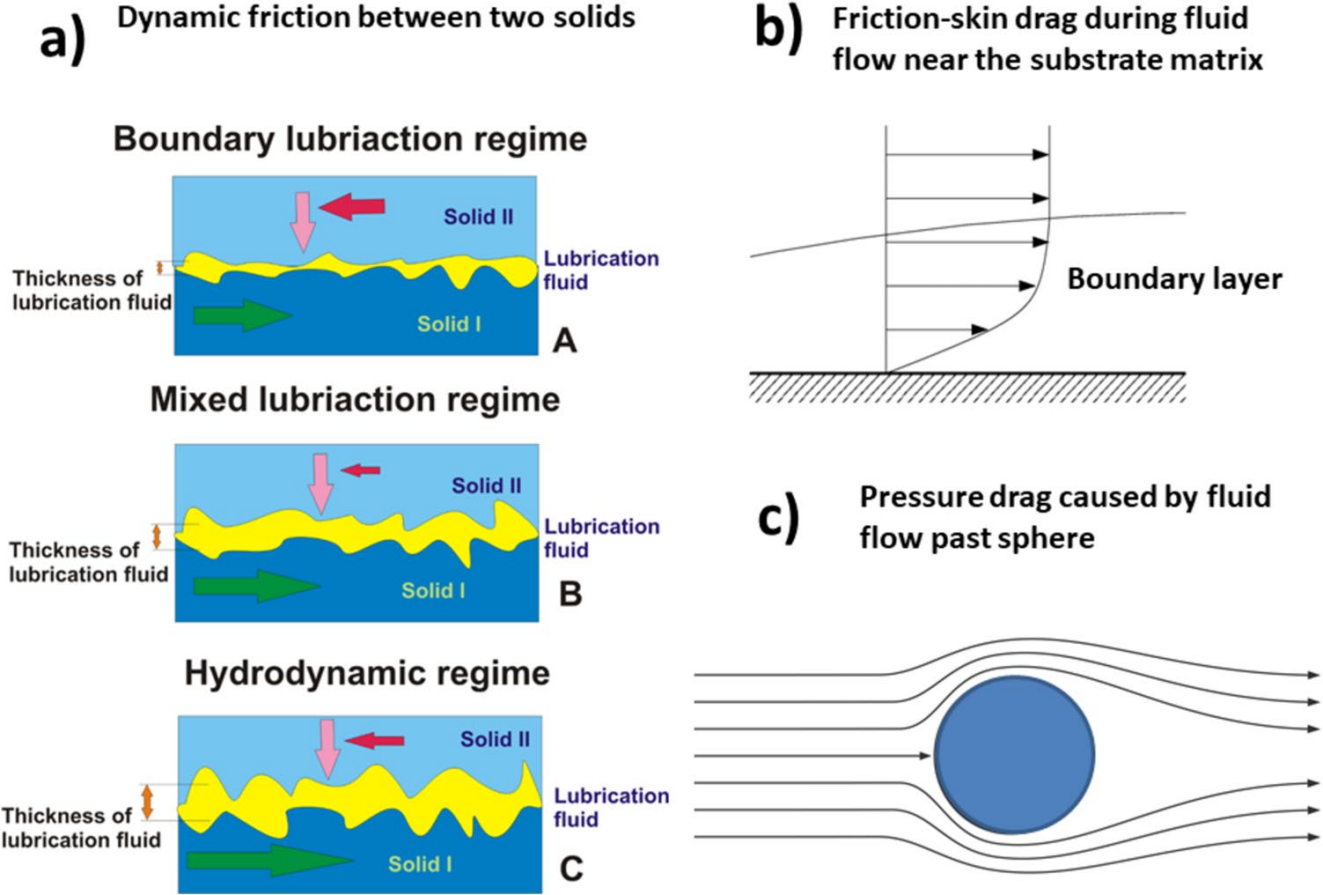
Schematic presentation of frictional effects generated within various types of systems: **a** dynamic friction between two solids considered within three regimes. Green arrows indicate the direction of relative velocity between two solid bodies, while pink arrows denote the direction of normal stress. Red arrows illustrate the direction of the frictional force. The maximum frictional force, in conjunction with the friction coefficient, is achieved in the boundary lubrication regime. Both of these parameters decrease during the mixing regime, followed by an increase in the hydrodynamic regime, all under consistent normal loading conditions. **b** Friction-skin drag caused by fluid flow along the solid wall, and **c** fluid flow past solid sphere/droplet

A diversity of different types of systems has been considered in the context of frictional effects, e.g., two solids in contact such as rocks or metals, rubber in contact with a metal surface, flow of polymer solutions along hydrophilic/hydrophobic solid surface (wall), flow of polymer solutions past a solid particle/droplet. The corresponding frictional effects have been quantified by dimensionless frictional coefficients such as (i) the dynamic friction coefficient, (ii) friction-skin drag, and (3) pressure drag, as shown schematically in Fig. 1.

In order to gain deeper insight into the role of viscoelasticity in the appearance of frictional effects, we now discuss the main theoretical approaches used.

### Dynamical friction between two solids

The relative motion between two solids along their interface area induces interface wear accompanied by viscoelastic and plastic structural changes, which cause energy dissipation. The wear relates to boundary lubrication conditions (i.e., high normal force, slow sliding velocity). Dynamic friction includes several distinct cases, depending on the surface roughness and stiffness ratio between solids 1 and 2 in contact expressed as $X=\frac{{E}_{1}}{{E}_{2}}$ (where ${E}_{1}$ and ${E}_{2}$ are Young’s moduli of the two solids) (Schallamach 1963; Al-Bender et al. 2005; Persson 2001). These cases are as follows:

- The occurrence of wear along the interface can be neglected when (i) solid 1 slides over a flat adhesive solid 2 or (ii) the Young’s moduli of solids 1 and 2 are such that ${E}_{2}>{E}_{1}$ (Schallamach 1963).
- Moderate wear occurs along the interface when (i) solid 1 slides over a rough solid 2 and (ii) the Young’s moduli of solids 1 and 2 are ${E}_{2}\sim {E}_{1}$ (Al-Bender et al. 2005).
- Intensive wear occurs on various space scales when (i) solid 1 slides over a rough solid 2 and (ii) the Young’s moduli of solids 1 and 2 are such that ${E}_{2}\gg {E}_{1}$ (Persson 2001).

The primary factor affecting dynamic friction in the first case is adhesion. Schallamach (1963) expressed the frictional force as

1 $${\overrightarrow{F}}_{F}=N{k}_{B}{\tau }_{B}\overrightarrow{v}$$

where $N$ is the number of bonds, ${k}_{B}$ is the spring constant per single bond, ${\tau }_{B}$ is the average bond lifetime, and $\overrightarrow{v}$ is the sliding velocity.

In the case of moderate wear, the structural changes can be described by one internal state variable $\overrightarrow{z}\left(r,\tau \right)$, which corresponds to the average deformation of surface asperities. Consequently, the frictional force ${\overrightarrow{F}}_{F}$ can be expressed as

2 $${\overrightarrow{F}}_{F}\left(r,\tau \right)={ {\widetilde{\sigma }}_{surf}}^{s}\bullet \overrightarrow{t}{ A}_{int}$$

where $r$ is the surface coordinate, $\tau$ is time, $\overrightarrow{t}$ is the unit tangential vector, ${A}_{int}$ is the interfacial area, and ${{\widetilde{\sigma }}_{surf}}^{s}\left(r,\tau \right)$ is the total shear stress along the interface, which includes elastic and viscous contributions caused by solid structural changes and hydrodynamic contribution caused by flow of a lubrication fluid. The frictional force caused by stick–slip motion can be expressed as ${\overrightarrow{F}}_{F}={\overrightarrow{F}}_{F}\left(\overrightarrow{z},\overrightarrow{v}\right)$ (where $\overrightarrow{v}\left(r,\tau \right)$ is the relative velocity between solids along the interface, and $\tau$ is time). Change of the internal state variable can be expressed as $\frac{d\overrightarrow{z}\left(r,\tau \right)}{d\tau }=g\left(\overrightarrow{z},\overrightarrow{v}\right)$ (where $g\left(\bullet \right)$ is the proper function which accounts for the surface viscoelasticity). The Lund and Grenoble formulation of the frictional force is one of the most widely used approaches for the modeling and simulation of dynamic frictional effects despite the fact that this model neglects the pre-sliding regime (Al-Bender et al. 2005). The so-called LuGre model is expressed as

3 $${\overrightarrow{F}}_{F}\left(r,\tau \right)={k}_{1}\overrightarrow{z}+{k}_{2}\frac{d\overrightarrow{z}}{d\tau }+{k}_{3}\overrightarrow{v}$$

where ${k}_{1}$ is the asperity spring constant, ${k}_{2}$ is the micro-viscous friction coefficient, and ${k}_{3}$ is the viscous friction coefficient accounting for the lubrication effects caused by fluid flow along the interface (Al-Bender et al. 2005). The first term on the right-hand side of Eq. 2 represents an elastic contribution, while the second and third parts account for viscous contributions to the friction force. The small perturbation of the velocity $\overrightarrow{{\varvec{v}}}$ from $\overrightarrow{{\varvec{v}}}={\overrightarrow{{\varvec{v}}}}_{0}$ to $\overrightarrow{{\varvec{v}}}={\overrightarrow{{\varvec{v}}}}_{0}+\Delta \overrightarrow{{\varvec{v}}}$ such that ${\overrightarrow{{\varvec{v}}}}_{0}\gg \Delta \overrightarrow{{\varvec{v}}}$ induces changes in the interfacial shear stress from the steady value ${\widetilde{{\varvec{\sigma}}}}_{{\varvec{s}}{\varvec{u}}{\varvec{r}}{\varvec{f}}\boldsymbol{ }{\varvec{s}}{\varvec{s}}}$ to the value ${{\widetilde{{\varvec{\sigma}}}}_{{\varvec{s}}{\varvec{u}}{\varvec{r}}{\varvec{f}}}}^{s}\left(r,\tau \right)$. The relaxation of stress can be expressed as (Rice and Ruina 1983)

4 $${{\widetilde{\sigma }}_{surf}}^{s}\left(r,\tau \right)\bullet \overrightarrow{t}={\widetilde{\sigma }}_{surf ss}\bullet \overrightarrow{t}+{k}_{3}\Delta \overrightarrow{v}\left(r,\tau \right)-{\int }_{0}^{t}h\left(r,\tau -{\tau }^{\prime}\right)\Delta \overrightarrow{v}\left(r,{\tau }^{\prime}\right)d{\tau }^{\prime}$$

where $h\left(r,\tau \right)$ is the memory kernel, which describes shear stress relaxation, ${\widetilde{\sigma }}_{surf ss}$ is the steady stress along the interface, and ${{\widetilde{\sigma }}_{surf}}^{s}\left(r,\tau \right)\bullet \overrightarrow{t}$ is the tangential traction force.

In the case of intensive wear, we will consider the sliding of elastic/hyper-elastic solids such as rubber on a rough metal surface. The altered structural changes occur on many length scales $l$ expressed by the distribution of strain ${\widetilde{\varepsilon }}_{surf}$ (Persson 2001). The phenomenon cannot be described by a single internal state variable. Rubber sliding induces fluctuations of the shear stress along the interface with the angular velocity $\omega =\frac{2\pi \Vert \overrightarrow{v}\Vert }{l}$ (where $\Vert \overrightarrow{v}\Vert$ is the speed). The shear stress ${{\widetilde{\sigma }}_{surf}}^{s}$ is equal to ${{\widetilde{\sigma }}_{surf}}^{s}={ {\widetilde{\sigma }}_{surf}}^{s}\left({{\widetilde{\varepsilon }}_{surf}}^{s}\right)$ (where ${{\widetilde{\varepsilon }}_{surf}}^{s}$ is the shear strain equal to ${{\widetilde{\varepsilon }}_{surf}}^{s}\left(r,\tau \right)=\frac{1}{2}\left(\overrightarrow{\nabla }\overrightarrow{u}+{\overrightarrow{\nabla }\overrightarrow{u}}^{T}\right)$ and $\overrightarrow{u}$ is the displacement field). The shear stress ${{\widetilde{\sigma }}_{surf}}^{s}$ accounts for the elastic and dissipative parts. A constitutive model ${{\widetilde{\sigma }}_{surf}}^{s}={ {\widetilde{\sigma }}_{surf}}^{s}\left({{\widetilde{\varepsilon }}_{surf}}^{s}\right)$ can be transformed into the frequency domain using the Fourier integral transform. The transformed equation can be expressed in the form $F\left[{ {\widetilde{\sigma }}_{surf}}^{s}\left(r,\tau \right)\right]={G}^{*}\left(r,\omega \right)F\left[{{\widetilde{\varepsilon }}_{surf}}^{s}\left(r,\tau \right)\right]$ (where $F\left[\bullet \right]$ is the Fourier transform, $\omega$ is the angular velocity, and ${G}^{*}\left(\omega \right)$ is the complex modulus). The complex modulus is equal to ${G}^{*}\left(r,\omega \right)={G}{\prime}\left(r,\omega \right)+i G"\left(r,\omega \right)$ (where ${G}{\prime}\left(r,\omega \right)$ is the storage modulus, $G"\left(r,\omega \right)$ is the loss modulus, and $i=\sqrt{-1}$ is the imaginary unit). The loss modulus $G"\left(r,\omega \right)$ is related to the internal friction of rubber along the interface area (Persson 2001). Viscoelastic energy dissipation within the bulk of the rubber can lead to the appearance of rolling friction along the interface (Butt and Kappl 2018).

The dimensionless dynamic frictional coefficient ${C}_{F}$ can be measured by tribometer, enabling physical parameters like the relative velocity $\overrightarrow{{\varvec{v}}}$ and the contact pressure $\Delta P$ to be externally chosen (Pitenis et al. 2018). It can be expressed as

5 $${C}_{F}=\frac{{ {\widetilde{\sigma }}_{surf}}^{s}}{{ {\widetilde{\sigma }}_{surf}}^{N}}$$

where ${{\widetilde{\sigma }}_{surf}}^{N}$ is the normal stress on the interface and ${\overrightarrow{n}\bullet {\widetilde{\sigma }}_{surf}}^{N}\bullet \overrightarrow{n}=\Delta P$ and $\Delta P$ is the contact pressure. Small contact pressure ensures elastic structural changes along the interface especially in the case of elastic solids such as rubber, while a contact pressure higher than the yield stress causes plastic strain of the solid (Butt and Kappl 2018). The dynamic frictional coefficient vs. relative velocity shows complex behavior, which can be discussed within three regimes. This coefficient is constant in the pre-sliding (i.e., dry friction) regime, then decreases in the transient regime, and increases again in the hydrodynamic regime (Stribeck curve) (Al-Bender et al. 2005).

Shear stress caused by the sliding of a hydrogel over a solid substrate can include three contributions: (i) a viscoelastic contribution within the boundary layer near the interface, (ii) a viscous contribution caused by the flow of a lubrication fluid, and (iii) interfacial (frictional) shear stress (Rennie et al. 2005).

While the frictional coefficient ${C}_{F}$ correlates shear stress with normal load (i.e., contact pressure), caused by relative motion of the two solids at their interface, the friction-skin drag correlates the shear stress component with inertial stress caused by fluid flow along the substrate matrix.

### Friction-skin drag caused by flow of polymer solutions along the solid wall

Motion of fluid along the substrate matrix induces frictional effects due to interactions between fluid molecules and the surface of the matrix, which can result in the adhesion of fluid components, fluid sliding, and backwards fluid flow over the matrix (Barnes 1995). The dimensionless friction-skin drag coefficient ${C}_{D}^{skin}$ depends on fluid inertial stress and shear stress expressed as

6 $${C}_{D}^{skin}=\frac{{ {\widetilde{\sigma }}_{surf}}^{s}}{{\widetilde{\sigma }}_{in}}$$

where ${{\widetilde{\sigma }}_{surf}}^{s}$ is the fluid shear stress, ${\widetilde{\sigma }}_{in}$ is the inertial stress, also known as the dynamic pressure, equal to ${\widetilde{\sigma }}_{in}=\frac{1}{2}{\rho }_{f}\overrightarrow{v}\otimes \overrightarrow{v}$, and ${\rho }_{f}$ is the density of the fluid. The drag force is equal to

7 $${\overrightarrow{F}}_{D}={\widetilde{\sigma }}_{in}\bullet \overrightarrow{t}{A}_{int}$$

where ${A}_{int}$ is the interfacial area. The frictional-skin drag of Newtonian fluids vs. ${R}_{e}$ number, i.e., Moody diagram, exhibits a discontinuity between the laminar and turbulent regimes. This coefficient decreases linearly in the laminar regime and non-linearly in the turbulent regime, depending on the interface roughness (Moody 1944).

We focus here on the flow of viscoelastic fluids such as polymer solutions. The local fluid velocity $\overrightarrow{v}$ can then be expressed as $\overrightarrow{v}=\frac{{\rho }_{l}}{\rho }{\overrightarrow{v}}_{l}+\frac{{\rho }_{p}}{\rho }{\overrightarrow{v}}_{p}$ (where ${\rho }_{l}$ is the density of the solvent, ${\rho }_{p}$ is the polymer density, and ${\rho }_{f}$ is the density of the fluid, i.e., ${\rho }_{f}={\rho }_{l}+{\rho }_{p}$) (Bird et al. 1977). The fluid stress along the rigid boundary ${\widetilde{\sigma }}_{surf}$ depends on the fluid viscoelasticity and can be expressed as ${\widetilde{\sigma }}_{surf}={ {\widetilde{\sigma }}_{surf}}^{s}+{ {\widetilde{\sigma }}_{surf}}^{N}$ (where ${{\widetilde{\sigma }}_{surf}}^{N}$ is the normal component of the stress). In the case of polymer solutions, the solvent behaves as a Newtonian fluid, while polymers show viscoelastic behavior caused primarily by inter- and intra-chain interactions. The corresponding stress ${\widetilde{\sigma }}_{surf}$ includes solvent and polymer contributions, i.e., ${\widetilde{\sigma }}_{surf}={{\widetilde{\sigma }}_{surf}}^{p}+{{\widetilde{\sigma }}_{surf S}}^{l}$ (where ${{\widetilde{\sigma }}_{surf S}}^{l}$ is the shear stress of solvent and ${{\widetilde{\sigma }}_{surf}}^{p}$ is the polymer stress equal to ${{\widetilde{\sigma }}_{surf}}^{p}={{\widetilde{\sigma }}_{surf N}}^{p}+{{\widetilde{\sigma }}_{surf S}}^{p}$, ${{\widetilde{\sigma }}_{surf N}}^{p}$ is the normal component of the polymer stress, and ${{\widetilde{\sigma }}_{surf S}}^{p}$ is its shear component). The normal component of the polymer stress is caused by polymer stretching, which is pronounced in circular Couette shear flow due to the generation of centrifugal force unlike the case of Poiseuille flow through pipes (Groisman and Steinberg 1998). The shear stress of Newtonian fluids, such as solvent, ${{\widetilde{\sigma }}_{surf S}}^{l}$ correlates linearly with the corresponding shear rate ${\dot{\widetilde{\varepsilon }}}_{lS}=\frac{1}{2}\left(\overrightarrow{\nabla }\overrightarrow{{v}_{l}}+{\overrightarrow{\nabla }\overrightarrow{{v}_{l}}}^{T}\right)$.

Although not physically accurate, the non-linear Oldroyd-B constitutive model, developed from the upper convected Maxwell model, has been established as the primary basis for nearly all complex flow calculations and analyses related to the viscoelasticity of dilute polymer solutions (Bird et al. 1977). The main characteristics of this non-linear constitutive model are that (i) polymer stress consists of elastic and viscous parts, (ii) stress can relax under constant strain rate, (iii) stress relaxation from its initial value towards the residual stress causes dissipation of the elastic part of the stress, (iv) the residual stress is purely dissipative, and (v) the strain cannot relax. The stress change and the corresponding change in strain rate occur almost on the same time scale, such that ${W}_{i}$ is in the range $1{\le W}_{i}<10$ (Groisman and Steinberg 1998; Varshney and Steinberg 2018).

While the friction coefficient ${C}_{D}^{skin}$ for viscous flow has been considered as function of the Reynolds number, i.e., ${C}_{D}^{skin}={C}_{D}^{skin}\left({R}_{e}\right)$, the coefficient ${C}_{D}^{skin}$ of viscoelastic fluids depends on both the Reynolds and Weissenberg numbers, i.e., ${C}_{D}^{skin}={C}_{D}^{skin}\left({R}_{e},{W}_{i}\right)$. In accordance with the fact that shear flow of a viscoelastic fluids causes a generation of normal stress accompanied by a shear stress component, it is possible to calculate the dynamical frictional coefficient besides the friction-skin drag.

Varshney and Steinberg (2018) considered the change of friction-skin drag during creep flow of polymer solutions, depending on the Reynolds and Weissenberg numbers, by keeping constant their ratio $El=\frac{{W}_{i}}{{R}_{e}}$ (where $El$ is the dimensionless elastic number). The friction-skin drag coefficient increases for lower ${W}_{i}$ and ${R}_{e}$ numbers, reaches a maximum, and then decreases again for higher values of ${W}_{i}$ and ${R}_{e}$ (Varshney and Steinberg 2018). The corresponding increase in the friction-skin drag coefficient can be induced by the stretching of polymer chains, while a decrease in the friction-skin drag coefficient can represent a consequence of orientation for already-stretched polymer chains in the direction of flow (Groisman and Steinberg 1998).

Fluid sliding along the substrate matrix reduces frictional effects. In the case of the flow of polymer solutions and other two-phase fluids, this phenomenon has been discussed in the context of the depletion of a dispersed phase from the matrix caused by an interplay between steric, hydrodynamic, viscoelastic, and chemical factors (Barnes 1995). This friction-reducing property has been exploited by using a hydrophobic substrate matrix for the flow of hydrophilic fluids (Fairhall and Garcıa-Mayoral 2018). The induced slip velocity depends on fluid viscoelasticity and can be formulated as a Navier-slip model for viscoelastic fluids. The model is expressed as

8 $${\overrightarrow{v}}_{slip}={k}_{s}{\left({ {\widetilde{\sigma }}_{surf}}^{s}\bullet \overrightarrow{t}\right)}^{m}$$

where ${\overrightarrow v}_{slip}$ is the slip velocity, ${k}_{s}$ is the slip coefficient, and $m$ is the slip exponent (Ferrás et al. 2017). Frictional effects caused by fluid flow along the substrate matrix are lower in comparison with the fractional losses caused by fluid flow past solid sphere/droplet (Southard 2023).

### Pressure drag caused by flow of polymer solutions past solid sphere

The dimensionless pressure drag coefficient is higher than that for friction-skin drag for flow of the same fluid, i.e., ${C}_{D}^{skin}\ll {C}_{D}^{press}$ (where ${C}_{D}^{press}$ is the pressure drag coefficient) (Southard 2023). This drag coefficient also depends on the relationship between the fluid shear and inertial stresses caused by flow past a solid sphere, i.e., ${C}_{D}^{press}=\frac{{ {\widetilde{\sigma }}_{surf}}^{s}}{{\widetilde{\sigma }}_{in}}$, where the frictional force in this case is equal to ${\overrightarrow{F}}_{D}={\widetilde{\sigma }}_{in}\bullet \overrightarrow{t}{A}_{H}$, ${A}_{H}$ is the projected surface normally to the direction of fluid flow equal to ${A}_{H}=\pi {r}_{H}^{2}$, and ${r}_{H}$ is the hydrodynamic radius of solid sphere/particle. In the case of laminar flow of a Newtonian fluid past a solid sphere, the pressure drag coefficient depends on the Reynolds number only and can be expressed as ${C}_{D}^{press}=\frac{24}{{R}_{e}}$. It means that the shear stress ${{\widetilde{\sigma }}_{surf}}^{s}$ is an order of magnitude higher than the inertial stress ${\widetilde{\sigma }}_{in}$ for laminar flow, while for turbulent flow ${{\widetilde{\sigma }}_{surf}}^{s}\sim {\widetilde{\sigma }}_{in}$. The corresponding frictional force for laminar flow is expressed as

9 $${\overrightarrow{F}}_{D}=\xi \overrightarrow{v}$$

where $\xi$ is the Stokes friction coefficient equal to $\xi =6\pi {\eta }_{c}{r}_{H}$. The pressure drag of Newtonian fluids decreases with the ${R}_{e}$ number within the laminar and transient flow regimes and becomes constant within the turbulent regime due to a change in the mechanism of momentum transfer from molecular to supramolecular.

For flow of a viscoelastic fluid past a solid sphere, the pressure drag coefficient depends on the Weissenberg and Reynolds numbers. Faroughi and Del Giudice (2022) reported that the pressure drag caused by flow of polymer solution past solid sphere can either increase or decrease for low ${W}_{i}$ number (i.e., ${W}_{i}<1$) depending on the magnitude of the ${R}_{e}$ number. Flow of polymer solution at low ${W}_{i}$ and ${R}_{e}$ numbers is symmetrical front-to-back causing a decrease in the pressure drag coefficient (Southard 2023). However, an increase in ${R}_{e}$ perturbs the flow symmetry even at low values of the ${W}_{i}$ number, leading to an increase in the pressure drag coefficient. An increase in ${W}_{i}$ causes an increase in the pressure drag for both lower and higher values of ${R}_{e}$. The presence of flexible polymer chains induces the appearance of turbulence at a lower ${R}_{e}$ number.

Slip effects, if they exist, are capable of reducing the pressure drag coefficient similarly to the case of the friction-skin drag coefficient (Berry et al. 2017). The flow of viscoelastic fluids past solid sphere is a simpler phenomenon than fluid flow past a droplet. In this case, the flow of external fluid causes a circular flow of internal fluid within the droplet.

### Pressure drag caused by flow of polymer solutions past droplet

When external fluid flows past a droplet and internal fluid within the droplet performs circular flow, it is necessary to take into consideration the frictional effects caused by the flow of both fluids. While the frictional effects of the external fluid can be described by a pressure drag coefficient, frictional effects caused by flow of internal fluid along the interface could be described by the frictional-skin drag coefficient. The flow fields of the internal and external fluids are inter-dependent according to the boundary conditions along the interface. For the no-slip condition, the external and internal velocities and stresses are equal along the interface. However, slip effects, if they exist, result in a discontinuity between the external and internal velocity fields and stresses (Dhar et al. 2020). The stress difference along the interface has been discussed in the form of the Marangoni stress caused by fluid flow from regions of lower to higher interfacial tension (Dhar et al. 2020).

When the external and internal fluids are viscoelastic, it is necessary to formulate the corresponding drag coefficients by introducing ${W}_{i}$ numbers for both fluids and more dimensionless numbers such as the Weber number ${W}_{e}=\frac{{\rho }_{i}{{v}_{d}}^{2}{D}_{d}}{{\gamma }_{ei}}$ (where ${\gamma }_{ei}$ is the interfacial tension between the external and internal fluids, ${v}_{d}$ is the droplet relative speed, ${\rho }_{i}$ is the density of the internal fluid, and ${D}_{d}$ is the droplet diameter) (Frohn and Roth 2000).

Frictional effects significantly influence the rearrangement of biological systems, as biological cells are very sensitive to such effects. Undesirable frictional effects are generated during physiological processes such as blood flow through blood vessels, tissue self-organization in morphogenesis, spreading of cancer, and collective cell migration on substrate matrix during wound healing. Cells use various mechanisms to protect themselves against frictional shear stress. Deeper insight into frictional effects along the biointerface between adjacent tissues and between tissue and a substrate matrix is needed to prevent or reduce cell damage.

## Frictional effects in biological systems

It is well known that frictional effects along the biointerface between adjacent tissues or tissue and a substrate matrix can (i) cause fragility of blood vessels, (ii) induce inflammation of epithelial cells that are in contact with hydrogel implants, (iii) reduce cell movement during wound healing, and (iv) influence morphogenesis and the spreading of cancer through epithelial tissues. Some types of cells, such as epithelial and endothelial cells, possess the capacity to control the shear stress to some extent. Cells also can develop mechanisms to protect themselves against undesirable frictional shear stress. In certain locations, these cells establish a rudimentary sensor network by producing soluble and surface proteins and engaging in interactions with receptor proteins. In our further consideration, we will discuss various frictional effects that appear in biological systems, emphasizing the possible consequences and discussing the cell’s self-protection mechanisms.

### Dynamic friction effects along the biointerface between epithelial cells and soft medical implants

**Fig. 2:**
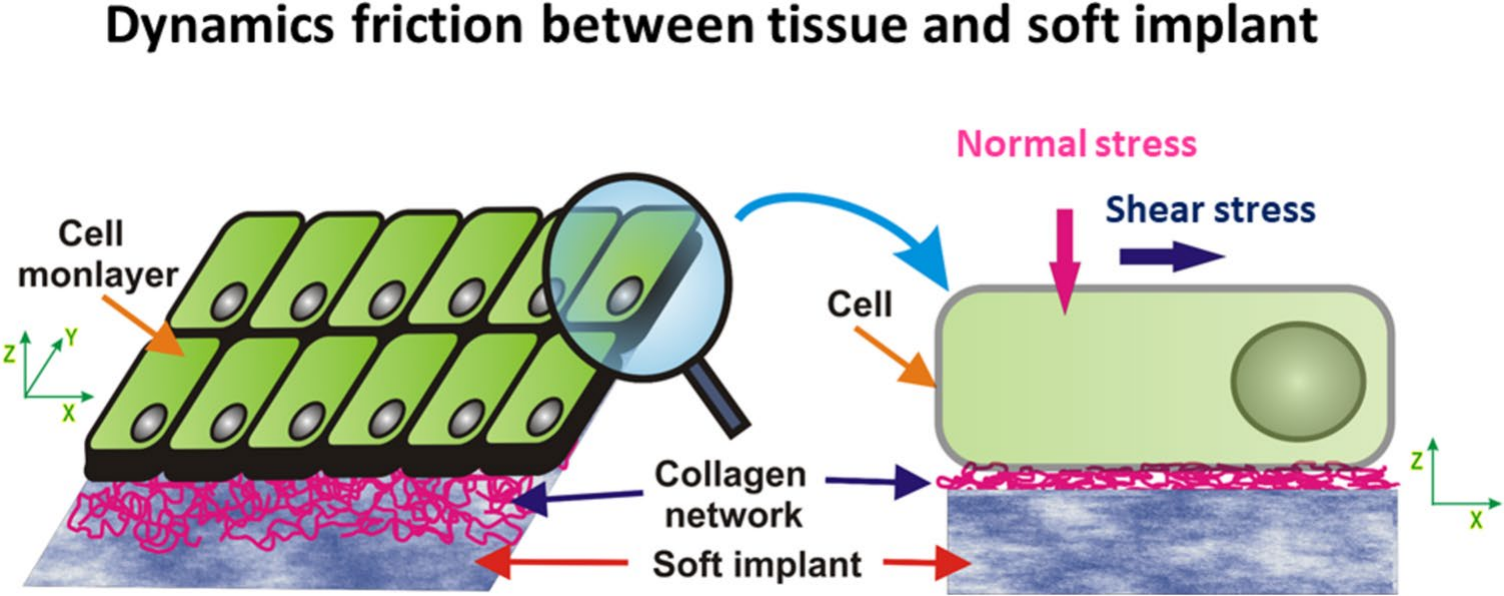
Frictional effects along the epithelial-implant biointerface may cause inflammation of the epithelial tissues resulting in fibrosis

Frictional shear stress of several tens of Pa can arise at the biointerface between epithelial cells and a soft medical implant formed from, e.g., silicone, elastomer, or polymer hydrogel. Contact lenses are a common example. In such cases, the shear stress impacts the behavior of neighboring cells. It is noteworthy that even minimal shear stress levels, below 1 Pa, have the potential to trigger a range of molecular responses including alterations in cell morphology, gene expression, cytoskeletal changes, modifications in cell–cell and cell–matrix adhesion, and in the case of epithelial cells, the initiation of the epithelial-to-mesenchymal transition (EMT) (Flitney et al. 2009; Delon et al. 2019; Pitenis and Sawyer 2020; Espina et al. 2023). Frictional effects along the epithelial-implant biointerface are shown schematically in Fig. 2.

The dynamic friction coefficient for viscoelastic soft matter systems such as epithelial tissues can be expressed as

10 $${C}_{Fe}=\frac{{\widetilde{\sigma }}_{Sre}+{\widetilde{\sigma }}_{fr}+{ {\widetilde{\sigma }}_{Sm}}^{vis}}{{ {\widetilde{\sigma }}_{surf}}^{N}}$$

where ${\widetilde{\sigma }}_{Sre}$ is the viscoelastic residual shear stress of epithelial tissue, ${\widetilde{\sigma }}_{fr}$ is the frictional shear stress, and ${{\widetilde{\sigma }}_{Sm}}^{vis}$ is the shear stress caused by flow of liquid medium along the biointerface. Espina et al. (2023) discussed various mechanisms of cell response under the shear stress, which has a feedback impact on the magnitude of the shear stress itself. The role of tissue viscoelasticity in epithelial sliding along the implant has not yet been investigated experimentally.

Frictional effects produced by the sliding of corneal epithelial cells along the biointerface with contact lenses at a speed of $250 \frac{\mu \text{m}}{\text{s}}$ under an externally induced contact pressure of $12 \text{kPa}$ pointed to boundary lubrication, while the corresponding dynamic friction coefficient was on the order of ${C}_{F}\sim 0.03$ (Angelini et al. 2012). This experimental setup allowed fine load control with low contact pressures and relatively low sliding speeds, thereby ensuring the suppression of hydrodynamic effects. In contrast to the lubrication caused by corneal epithelial cells’ friction with lenses, the friction and lubricity in the eye that occurs during a blink has been discussed within the context of hydrodynamic lubrication.

Pitenis et al. (2018) considered the response of human corneal epithelial (hTCEpi) cell monolayers under shear stress generated at the biointerface with hydrogel made with 7.5 wt% polyacrylamide and 0.3 wt% bisacrylamide. The corresponding dynamic friction coefficient was ${C}_{F}\sim 0.01$ (Chau et al. 2020). Pitenis et al. (2018) revealed that a shear stress of *60* Pa is capable of inducing inflammation of cells within 5.5 h. A higher dynamic friction coefficient could induce cell apoptosis.

Inflamed cells, caused by friction within a mixed lubrication regime, are stimulated to produce a fibrous capsule along the biointerface with the implant, i.e., a dynamic structure composed of dense connective fibrous tissues (Noskovicova et al. 2021). The immune response of soft tissues depends on the physical characteristics of implants. Doloff et al. (2021) revealed that the surface topography of silicone implants significantly influenced the immune response of breast tissue. The presence of such an undesirable fibrous capsule reduces the transport of nutrients towards the tissue, thereby enhancing cell apoptosis. Besides frictional effects caused by the contact of resting epithelial tissues with the soft implant, migrating epithelial tissues on substrate matrices also induce frictional effects. These can be discussed in the form of dynamic friction.

### Dynamic friction effects along the biointerface between collectively migrated epithelial monolayers on hydrogel substrate matrices

The collective migration of epithelial monolayers on substrate matrices also induces dynamic frictional effects. In this case, the sliding speed is significantly lower than the speed generated between tissue and soft implants, described in the previous section, i.e., $\le 1 \frac{\mu m}{min}$ (Serra-Picamal et al. 2012; Notbohm et al. 2016). Contact pressure is low and arises only as a consequence of the z-component of cell-substrate traction. The friction experienced by individual cells is a result of both internal and external factors. Internally, the friction is influenced by the actin retrograde flow, whereas externally, it is dependent on the strength of cell–matrix adhesion contacts (Ron et al. 2020). Cell–matrix adhesion contacts at the front of the cell go through a nucleation-maturation process, while those at the back experience successive attachment-detachment due to mechanical pulling (Broussard et al. 2008). Protrusive motion at the cell front can lead to stick–slip friction, which is associated with overall cell elongation (Ron et al. 2020). When considering cell monolayers, dynamic friction is viewed as the cumulative effects of external friction, discussed in the context of cell–matrix adhesion, while the wear along the biointerface is often disregarded (Vazquez et al. 2022). Vazquez et al. (2022) considered the dynamic friction by applying the model proposed by Schallamach (1963), discussed in Sect. 2.1, and concluded that an increase of substrate stiffness causes an increase in the number of cell–matrix bonds and bond strength, resulting in an increase in the dynamic friction. Jipp et al. (2024) pointed to the interplay between cell-substrate distance fluctuations, the strength of cell-substrate adhesion, and traction forces, which influence cell velocity and on that basis frictional effects.

**Fig. 3:**
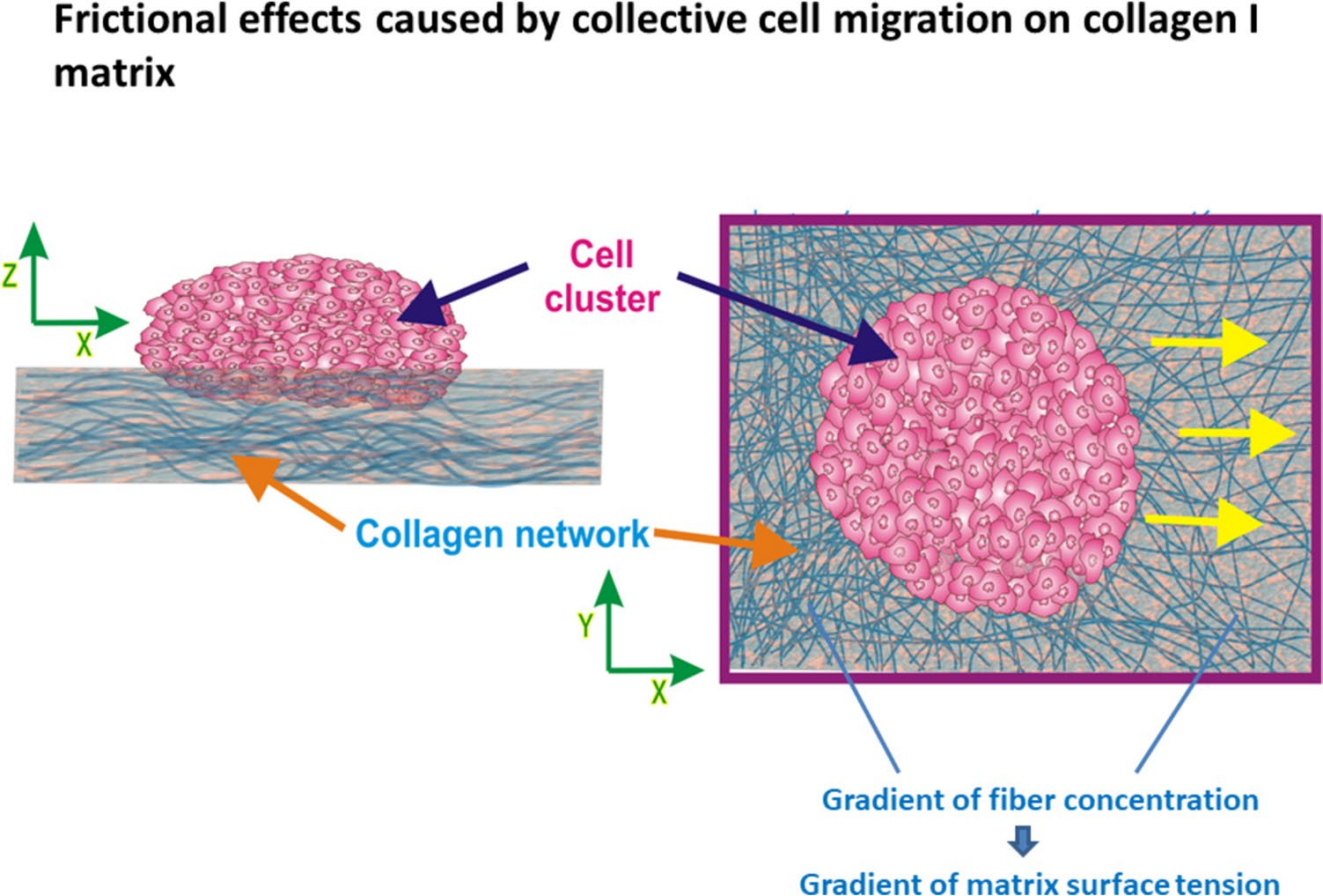
Schematic illustration of frictional effects on a substrate matrix caused by collective cell migration, where red arrows represent the direction of the cluster movement. The surface concentration of collagen fibers in front of the cluster is lower than the concentration just behind it, in accordance with the experimental findings of Clark et al. (2022). (Yellow arrows represent a direction of cluster movement.)

However, cell movement on a substrate matrix can induce wearing of the matrix, which has an impact on the strength of the cell–matrix adhesion contacts accompanied by the dynamic friction as shown in Fig. 3.

The migration of clusters induces surface structural modifications in the collagen I matrix, leading to the extension of collagen fibers and their radial alignment with the cell cluster. These changes cause variations in collagen concentration around the cell cluster. Consequently, the distribution of collagen concentration around the cell cluster exhibits an asymmetry, with the collagen levels being approximately 30% lower near the front of the cluster compared to the rear (Clark et al. 2022). While frictional effects caused by collective migration of epithelial monolayers on substrate matrices have been discussed in the context of the dynamic friction coefficient, blood flow through blood vessels has been discussed in the context of friction-skin drag.

### Friction-skin drag caused by blood flow along blood vessels

Blood is a viscoelastic fluid consisting of blood cells, such as erythrocytes, leucocytes, and platelets, in an aqueous plasma phase containing dissolved proteins. The shear strain rate caused by the blood flow differs widely between the large arteries, the veins, and the capillaries, and it varies from a few ${\text{s}}^{-1}$ up to $1000 {\text{s}}^{-1}$ (Connes et al. 2016). The viscoelasticity of blood depends on several factors: (i) the concentration and deformability of erythrocytes in the flow, (ii) the ability of erythrocytes to form aggregates (rouleaux) and their disaggregation in flow, and (iii) the viscoelasticity of blood plasma (Baskurt and Meiselman 2003). Variants of the Maxwell, Jeffreys, and Oldroyd-B constitutive models have been used for describing the viscoelasticity of blood (Armstrong et al. 2018). While the Maxwell and Jeffreys models are linear, the Oldroyd-B model is non-linear. None of these models is suitable for describing structural changes of blood under low shear rate $<0.1 {s}^{-1}$. Their main characteristics are that (i) stress can relax under constant strain rate, (ii) the strain rate can relax under constant stress conditions only for the Jeffreys model, and (iii) the corresponding Weissenberg number is in the range of ${W}_{i}\sim 1-10$ (Armstrong et al. 2018; Pajic-Lijakovic et al. 2024a).

The friction-skin drag coefficient has been calculated based on empirical correlations as a function of the Reynolds number, while the Weissenberg number has not been included explicitly (Chapman and Cokelet 1998; Yilmaz et al. 2011).

The frictional effects along blood vessels are influenced by the hydrodynamic blood shear stress, which is dependent on factors such as blood velocity, viscoelasticity, and the diameter of the blood vessel. The physiological shear stress induced by blood flow, ranging from 1 to 5 Pa, creates an optimal physiological setting for endothelial monolayers in vivo. This environment encourages cell elongation and polarity-flow alignment, while inhibiting proliferation, enhancing the expression of anti-inflammatory genes, and reducing the expression of inflammatory pathways.

Cells exhibit sensitivity to slight variations in both the magnitude and direction of blood flow-induced shear stress, as well as to perturbations in this stress (Givens and Tzima 2016). Alterations in shear stress can impact cell morphology, blood vessel permeability, and cell–cell adhesion contacts, and they may trigger inflammatory responses. Higher or perturbed shear stress has the potential to initiate the development of vascular conditions like atherosclerosis and aneurysms (Cunningham and Gotlieb 2005). Cells possess the ability to regulate the shear stress of blood to a certain degree (Roux et al. 2020). Consequently, a localized reduction in shear stress can result in (1) cell movement against the blood flow and (2) alterations in cell shape and adhesion contacts, leading to a thinning of the cell monolayer and a subsequent reduction in blood vessel diameter (Langille et al. 1989). These cellular activities result in an increase in the shear stress. Shear stress ranging from 0.5 to 1 Pa has a significant impact on both the structure and function of endothelial cells. This includes elongation and alignment of the cells along the flow, reorganization of the F-actin network, and adjustment of cell stiffness (Park et al. 2011). In a study by Conklin et al. (2007), porcine carotid arterial endothelial cells were exposed to low shear stress of 0.15 Pa and physiological shear stress. It was observed that low shear stress caused a 2.8-fold increase in blood vessel permeability due to the weakening of tight junctions (TJs) after 12 h. Steward et al. (2015) investigated the impact of 1.2 Pa shear stress on cell–cell adhesion contacts in a collectively migrating endothelial monolayer. They found that this level of shear stress led to the weakening of adherens junctions (AJs), resulting in a reduction of inter-cellular normal stress after 12 h.

**Fig. 4:**
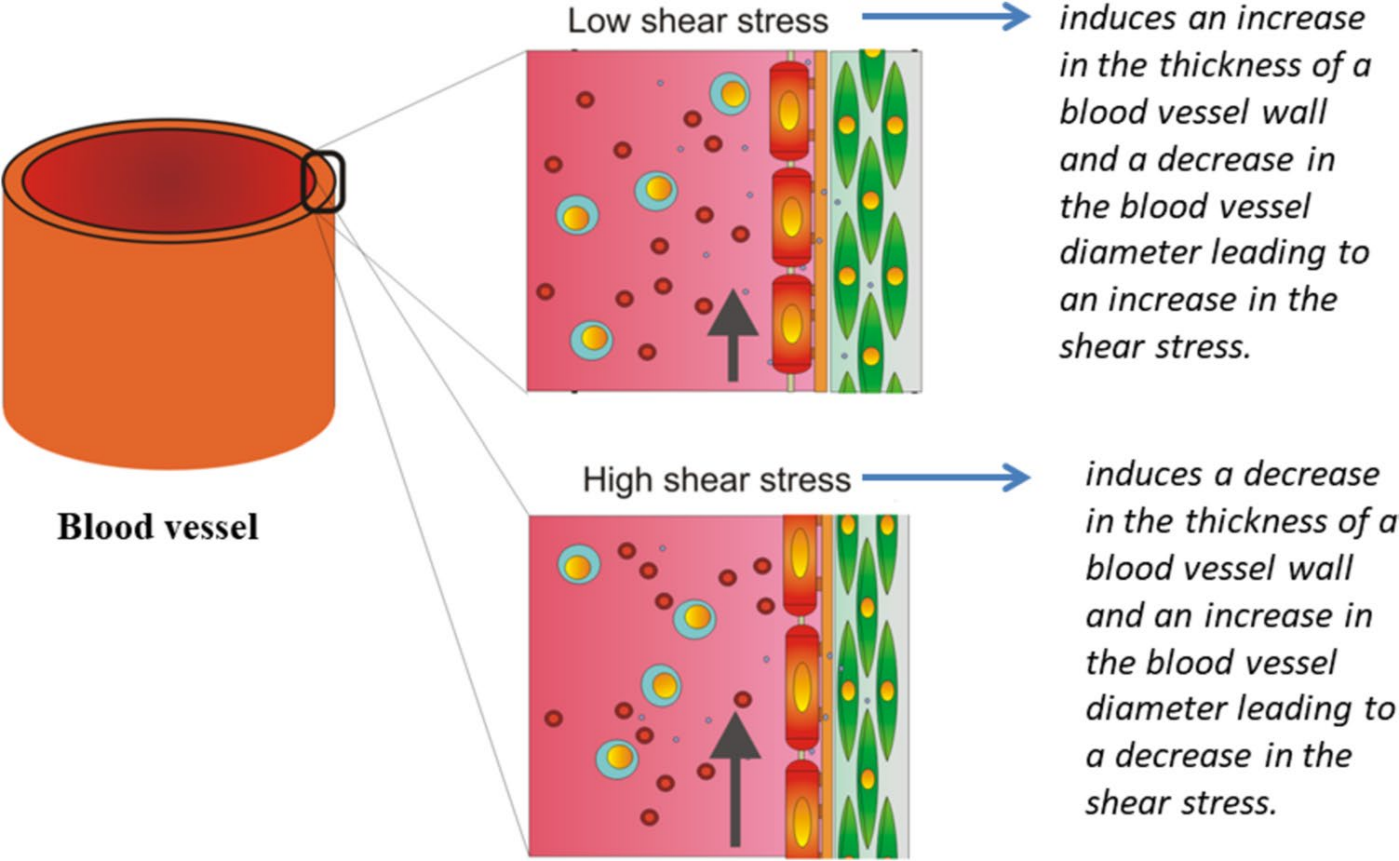
Endothelial response under various shear stress caused by blood flow (Black arrows represent the direction of blood flow through blood vessels)

Endothelial cells are able to self-regulate the shear stress caused by blood flow through blood vessels as shown in Fig. 4.

Otherwise, a localized increase in shear stress results in (1) cellular movement in the direction of blood flow and (2) alterations in cell shape and adhesion contacts, leading to a thickening of the cell monolayer and correspondingly an enlargement in the diameter of the blood vessel. These cellular responses contribute to a reduction in shear stress. Endothelial cells subjected to low shear conditions of ≤ 1 Pa within blood vessels exhibit fewer central stress fibers compared to cells under normal, unstressed conditions, as a protective mechanism against localized cell disintegration. Conversely, cells exposed to high shear conditions (several tens of Pa) experience an increase in thickness and length of central microfilaments, while the peripheral ones undergo disruption (Colangelo et al. 1998). The reorganization of the cytoskeleton under blood shear stress is primarily triggered by the phosphorylation of the intermediate filament network (Flitney et al. 2009).

While frictional effects caused by blood flow through blood vessels can be described by the friction-skin drag coefficient, frictional effects caused by collective migration of cancer cells past epithelial clusters can better be described by the pressure drag coefficient, which will be discussed in the next section.

### Frictional effects caused by collective migration of *cancer* cells past epithelial clusters in co-cultured epithelial-*cancer* spheroids

Frictional effects along the biointerface between epithelial and mesenchymal-like cancer cells occur within co-cultured spheroids during the process of segregation. Epithelial cells migrate in the form of strongly connected clusters towards the spheroid core region, while cancer cells migrate as a stream in the opposite direction towards the surface of the co-cultured epithelial-cancer spheroids. Consequently, the epithelial subpopulation represents a dispersed phase, while the cancer subpopulation is a continuous phase. The segregation process is driven by the interplay between the epithelial-cancer interfacial tension, the interfacial tension gradient, and collective cell migration (Pajic-Lijakovic et al. 2023). The interfacial tension can be expressed as (Pajic-Lijakovic et al. 2024a)

11 $${\gamma }_{ce}={\gamma }_{c}+{\gamma }_{e}-{e}_{a}$$

where ${\gamma }_{c}$ and ${\gamma }_{e}$ are the surface tensions of the cancer and epithelial subpopulations in contact with the liquid medium, and ${e}_{a}$ is the adhesion energy, which depends on heterotypic epithelial-cancer interactions. The surface tensions of the subpopulations rely on the strength of cell–cell adhesion contacts and cell contractility. While epithelial cells establish strong E-cadherin-mediated cell–cell adhesion contacts, mesenchymal-like cancer cells establish weak cell–cell adhesion contacts (Devanny et al. 2021). Consequently, active contractile cells satisfy the condition that ${\gamma }_{c}\ll {\gamma }_{e}$ (Devanny et al. 2021). The ability of cells to form heterotypic cell–cell adhesion contacts causes a decrease in the interfacial tension.

The interfacial tension exerts work on a decrease in the biointerface area by compressing the epithelial subpopulation and extending the cancer subpopulation. Higher interfacial tension ensures more efficient cell segregation (Lucia et al. 2022; Pajic-Lijakovic et al. 2024b). In this context, two segregation scenarios are possible: (i) complete segregation and (ii) partial segregation. Complete segregation occurs when the epithelial subpopulation forms a single large cluster in the core region, while the cancer subpopulation arrives at the surface region of the spheroid. In this case, the epithelial-cancer biointerface is minimal. For lower interfacial tension, the epithelial subpopulation forms smaller dispersed clusters within the cancer subpopulation.

Interfacial tension is in-homogeneously distributed along the biointerface caused by heterotypic cell–cell interactions, which also have a feedback impact of homotypic cell–cell interactions within the epithelial subpopulation (Pajic-Lijakovic et al. 2024a). Consequently, the gradient of the interfacial tension influences cell movement along the biointerface from the regions of lower interfacial tension to those of higher interfacial tension (Pajic-Lijakovic et al. 2023). The phenomenon is known as the Marangoni effect (Pajic-Lijakovic et al. 2023). The Marangoni effect has been confirmed experimentally by Gsell et al. (2023).

**Fig. 5:**
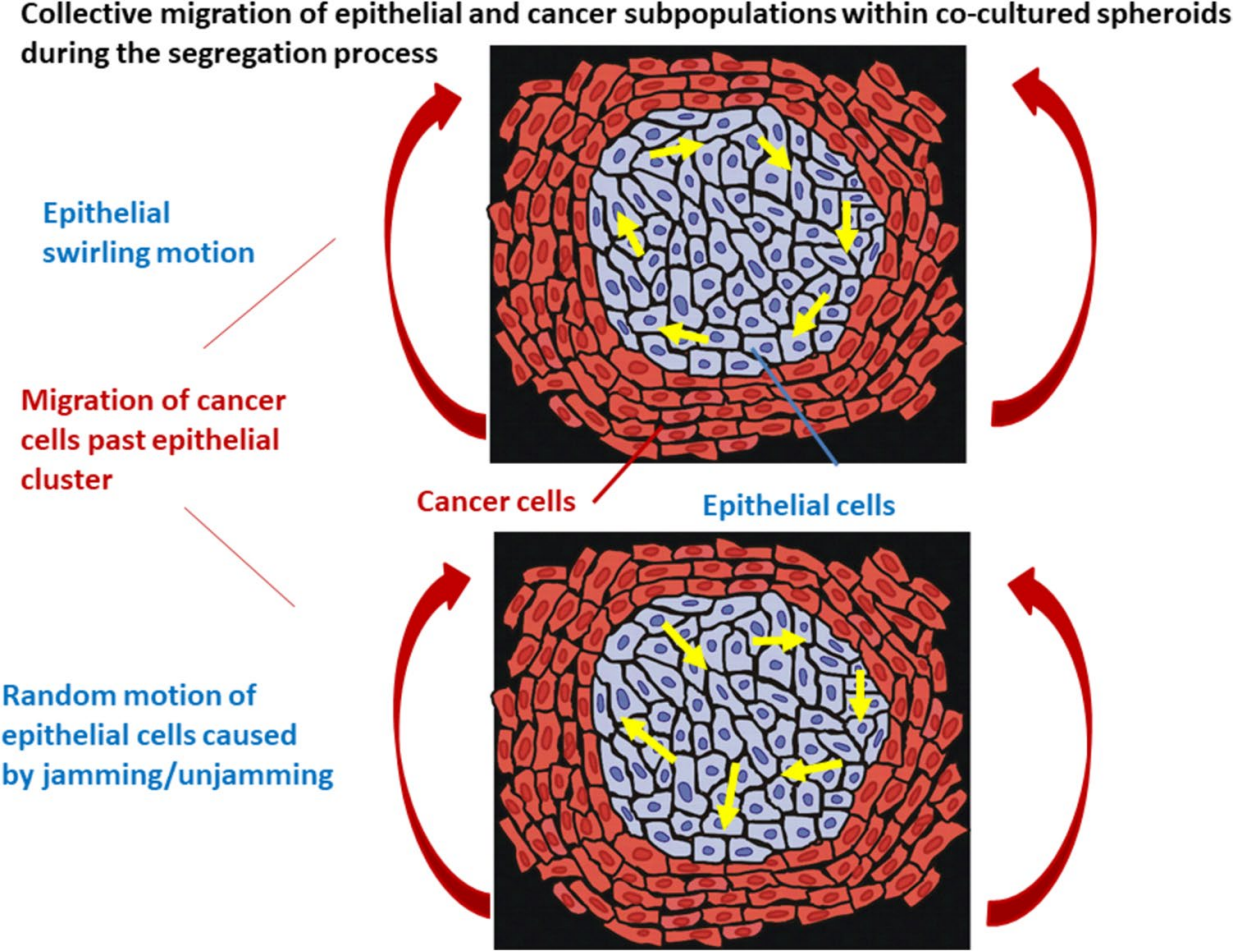
Collective migration of epithelial and cancer subpopulation within co-cultured spheroids under in vitro conditions during the process of segregation (Red arrows represent the direction of cancer cell migration, while yellow arrows represent two different modes of epithelial cell migration within the cluster)

When cancer cells migrate through epithelial clusters, two distinct modes of movement can be observed in epithelial cells within the clusters. These modes, triggered by the build-up of compressive stress, are (i) random movement resulting from the repeated transition between cell jamming and unjamming and (ii) swirling motion of cells caused by the contraction of an already established supracellular actin network along the clusters. (Lucia et al. 2022; Pajic-Lijakovic et al. 2023) as was shown in Fig. 5.

Both modes of epithelial migration represent parts of epithelial tendency to protect themselves against undesirable shear and compressive stress components.

- Cell jamming is caused by an increase in epithelial packing density, which intensifies cell–cell interactions and on that basis causes the contact inhibition of locomotion (CIL). The CIL leads to weakening of cell–cell adhesion contacts and cell repolarization (Roycraft and Mayor 2016; Zimmermann et al. 2016). When the average time between two collisions is shorter than the cell repolarisation time, cells undergo jamming, i.e., the contractile-to-non-contractile cell state transition (Pajic-Lijakovic and Milivojevic 2022). Weakening of E-cadherin mediated cell–cell adhesion contacts, caused by cell jamming, induces energy dissipation leading to a decrease in the shear and compressive stress components.
- Weakening of cell–cell adhesion contact can induce single cell rolling under frictional shear stress resulting in an additional decrease in the frictional effects along the epithelial-cancer biointerface.
- In some cases, epithelial cells form a supracellular actin network along the biointerface with cancer cells (Lucia et al. 2022). This actin network serves as a shell around the cluster, which causes stiffening of the epithelial cells along the biointerface and, consequently, an increase in the resistance effects and minimization of the wear. Contractions of the supracellular network can induce cell swirling motion accompanied by successive radial pulsation of epithelial clusters (Lucia et al. 2022; Notbohm et al. 2016; Pajic-Lijakovic et al. 2024a).

Lucia et al. (2022) considered segregation of co-cultured monolayers and pointed out that epithelial Madin-Darby canine kidney type II (MDCK) cells undergo random migration within clusters surrounded by cancer C2C12 cells. However, epithelial HaCaT cells show quite different behavior in contact with cancer C2C12 cells. In particular, the epithelial cells form a supracellular actin network along the biointerface with the cancer cells.

Frictional effects between the epithelial and cancer subpopulations along the biointerface depend on the viscoelasticity of subpopulations. Viscoelasticity of the subpopulations relies on the strength of cell–cell adhesion contacts and cell contractility (Pajic-Lijakovic et al. 2024b). A suitable constitutive model for the epithelial subpopulation was proposed based on experimental findings from Serra-Picamal et al. (2012) and Notbohm et al. (2016). They pointed out that cell residual stress correlates with the corresponding strain. Khalilgharibi et al. (2019) discussed the ability of epithelial monolayers to relax under constant strain conditions. In this line, the viscoelasticity of the epithelial subpopulation corresponds to that of a viscoelastic solid and has therefore been described by the Zener model. Its main characteristics are that (i) stress can relax under constant strain conditions, (ii) strain can relax under constant stress condition, and (iii) the residual stress is elastic. In this case, the stress relaxes through many short-time relaxation cycles under constant strain per cycle (Pajic-Lijakovic et al. 2024b).

In accordance with the fact that cancer cells establish weak cell–cell adhesion contacts and migrate as a stream, Pajic-Lijakovic et al. (2022) proposed the use of the Maxwell model, suitable for viscoelastic liquids, for describing the viscoelasticity caused by the collective migration of the cancer subpopulation. The applicability of the Maxwell model has been confirmed through experiments on cell aggregate micropipette aspiration (Guevorkian et al. 2021). Intensive energy dissipation caused by the disruption of cell–cell bonds during cell movement towards the pipette leads to liquid-like behavior. The main characteristics of the Maxwell model in this application are that (i) stress can relax under constant strain rate conditions, (ii) strain cannot relax, (iii) the residual stress is purely dissipative, (iv) the stress relaxation time corresponds to a time scale of minutes, while the strain change occurs on a time scale of hours. Both of these constitutive models are presented in the Appendix.

Consequently, stress relaxes through a sequence of short-time relaxation cycles under constant strain rate per cycle (Pajic-Lijakovic and Milivojevic 2022). The viscoelastic contribution to the cell shear stress generated during the collective migration of epithelial monolayers corresponds to several tens of Pa (Tambe et al. 2013; Serra-Picamal et al. 2012). The inertial stress caused by movement of epithelial/cancer subpopulations is much lower, i.e.,$\ll 1 \text{Pa}$. This is in accordance with the fact that the cell velocity is low, $\le 1 \frac{\mu m}{min}$ (Clark and Vignjevic 2015). The velocity of cancer cells is higher than that of epithelial cells under the same cell packing density (Clark and Vignjevic 2015). Consequently, the Weissenberg numbers of the subpopulations satisfy the condition that ${W}_{i}^{e}<{W}_{i}^{c}$ and ${W}_{i}^{e} ,{W}_{i}^{c}\ll 1$ (where ${W}_{i}^{e}$ and ${W}_{i}^{c}$ are the epithelial and cancer Weissenberg numbers, respectively). It does not mean that the cancer subpopulation is more elastic; rather, it means that the rate of strain change of the migrating cancer subpopulation is higher than that of the epithelial subpopulation, while the stress relaxation time in both cases corresponds to a time scale of minutes.

The long-time viscoelasticity of the subpopulations, caused by collective cell migration, is connected to the behavior of the cell residual stress. It means that the epithelial subpopulation behaves as an elastic solid, while the cancer subpopulation behaves as a Newtonian liquid (Pajic-Lijakovic and Milivojevic 2022).

Frictional effects caused by the movement of cancer cells past epithelial cluster can be treated in terms of Stokes law, formulated for Newtonian fluids and described by the effective pressure drag for the low Reynolds number, i.e., ${C}_{Dc}^{press}=\frac{24}{{R}_{e c}}$ (where ${R}_{e c}=\frac{{\rho }_{c}U{d}_{H}}{{\upeta }_{c}}$ is ${d}_{H}$ is the hydrodynamic radius of the epithelial cluster,${\rho }_{c}$ is the density of the cancer subpopulation, and ${\upeta }_{c}$ is the cancer viscosity).

The presence of the cancer subpopulation around the epithelial cluster can influence the state of the cluster itself.

Friction caused by the movement of epithelial cells depends on the epithelial shear stress along the biointerface and the compressive stress generated as a product of collective cell migration (the deviatoric part of the stress) and the work done by the interfacial tension (the isotropic part of the stress). Epithelial shear stress includes three contributions: (i) shear stress caused by viscoelasticity of the epithelial subpopulation; (2) frictional shear stress, accounting for effects from sliding along the biointerface; and (iii) hydrodynamic shear stress caused by flow of the liquid medium.

The epithelial friction in this type of system has not yet been discussed, but could be interpreted as the dynamic friction coefficient:

12 $${C}_{Fe}=\frac{{\widetilde{\sigma }}_{Sre}+{\widetilde{\sigma }}_{fr}+{ {\widetilde{\sigma }}_{Sm}}^{vis}}{\Delta {\widetilde{\sigma }}_{Nr}}$$

where $\Delta {\widetilde{\sigma }}_{Nr}$ is the normal residual stress difference between epithelial and cancer subpopulations along the biointerface, ${\widetilde{\sigma }}_{Sre}$ is the epithelial shear residual stress, ${\widetilde{\sigma }}_{fr}$ is the frictional shear stress along the biointerface, and ${{\widetilde{\sigma }}_{Sm}}^{vis}$ is the shear stress caused by flow of the liquid medium, which serves as a lubricant. All stress contributions are expressed in Table 1.

**Table 1:** Various contributions to the shear and normal stresses along the biointerface, which influence the frictional effects exhibited by epithelial cells

Normal/shear stress contributions	Model equation
Normal residual stress difference along the biointerface ${\Delta \widetilde{\sigma }}_{rN}$ (Pajic-Lijakovic et al. 2023)	${\Delta \widetilde{\sigma }}_{rN}=-\Delta {p}_{c\to e}\widetilde{I}+\Delta {{\widetilde{\sigma }}_{rN}}^{CCM}$ $\Delta {p}_{c\to e}=-{\gamma }_{ce }\left(\overrightarrow{\nabla }\bullet \overrightarrow{n}\right)$ is the isotropic part of the normal stress $\Delta {{\widetilde{\sigma }}_{rN}}^{CCM}$ is the deviatoric part of the normal stress difference $\Delta {{\widetilde{\sigma }}_{rN}}^{CCM }={{\widetilde{\sigma }}_{erN}}^{CCM }-{{\widetilde{\sigma }}_{crN}}^{CCM}$ ${{\widetilde{\sigma }}_{erN}}^{CCM}$ is the normal residual stress of epithelial subpopulation ${{\widetilde{\sigma }}_{crN}}^{CCM}$ is the normal residual stress of cancer subpopulation
Normal residual stress of the epithelial subpopulation caused by collective cell migration ${{\widetilde{\sigma }}_{rNe}}^{CCM}$(Pajic-Lijakovic et al. 2023)	${{\widetilde{\sigma }}_{rNe}}^{CCM}={E}_{e} {\widetilde{\varepsilon }}_{eN}$ where ${E}_{e}$ is the normal elastic modulus of the epithelial subpopulation, ${\widetilde{\varepsilon }}_{e}$ is the normal strain equal to ${\widetilde{\varepsilon }}_{eN}=\overrightarrow{(\nabla }\bullet {\overrightarrow{u}}_{e})\widetilde{I}$, ${\overrightarrow{u}}_{e}$ is the displacement field of epithelial cells, and $\widetilde{I}$ is the unit tensor
Normal residual stress of the cancer subpopulation caused by collective cell migration ${{\widetilde{\sigma }}_{rNc}}^{CCM}$(Pajic-Lijakovic et al. 2023)	${{{\widetilde{\sigma }}_{crN}}^{CCM}=\eta }_{c}{\dot{\widetilde{\varepsilon }}}_{cN}$ where ${\eta }_{c}$ is the bulk viscosity of the cancer subpopulation, ${\dot{\widetilde{\varepsilon }}}_{Nc}$ is the normal strain rate of the cancer subpopulation equal to ${\dot{\widetilde{\varepsilon }}}_{cN}=\frac{d{\widetilde{\varepsilon }}_{cN}}{d{\tau }_{L}}$, ${\widetilde{\varepsilon }}_{cN}=\overrightarrow{(\nabla }\bullet {\overrightarrow{u}}_{c})\widetilde{I}$, and ${\overrightarrow{u}}_{c}$ is the displacement field of cancer cells
Shear stress of the epithelial subpopulation along the biointerface ${\widetilde{\sigma }}_{erS}$ (Pajic-Lijakovic and Milivojevic 2023)	$\overrightarrow{n}\bullet {\widetilde{\sigma }}_{erS}\bullet \overrightarrow{t}=\overrightarrow{\nabla }{\gamma }_{ce}\bullet \overrightarrow{t}+\overrightarrow{n}\bullet {{\widetilde{\sigma }}_{erS}}^{CCM }\bullet \overrightarrow{t}$ $\overrightarrow{\nabla }{\gamma }_{ce}$ is the gradient of interfacial tension ${{\widetilde{\sigma }}_{erS}}^{CCM}$ is the epithelial residual shear stress caused by collective cell migration
Shear stress of the epithelial subpopulation cussed by collective cell migration ${{\widetilde{\sigma }}_{erS}}^{CCM}$(Pajic-Lijakovic et al. 2023)	${{\widetilde{\sigma }}_{erS}}^{CCM}={G}_{e} {\widetilde{\varepsilon }}_{eS}$ where ${G}_{e}$ is the shear elastic modulus and ${\widetilde{\varepsilon }}_{Se}$ is the shear strain of epithelial subpopulation equal to ${\widetilde{\varepsilon }}_{eS}=\frac{1}{2}\left(\overrightarrow{\nabla }{\overrightarrow{u}}_{e}+{\overrightarrow{\nabla }{\overrightarrow{u}}_{e}}^{T}\right)$
Frictional shear stress ${\widetilde{\sigma }}_{fr}$ (Ferrás et al. 2017)	$\overrightarrow{n}\bullet {\widetilde{\sigma }}_{fr}\bullet \overrightarrow{t}={k}_{F}{\Vert {\overrightarrow{v}}_{slip}\Vert }^{m}$ where ${\overrightarrow{v}}_{slip}$ is the slip velocity equal to ${\overrightarrow{v}}_{slip}={\overrightarrow{v}}_{e}-{\overrightarrow{v}}_{c}$, ${\overrightarrow{v}}_{e}$ and ${\overrightarrow{v}}_{c}$ are velocities of epithelial and cancer subpopulations, respectively, ${k}_{F}$ is the measure of heterotypic cell–cell interactions along the biointerface, and $m$ is the exponent
Shear stress of a lubricating medium along the biointerface ${{\widetilde{\sigma }}_{Sm}}^{vis}$ (Rennie et al. 2005)	${{{\widetilde{\sigma }}_{Sm}}^{vis}=\eta }_{m}{\dot{\widetilde{\varepsilon }}}_{mS}$ where ${\eta }_{m}$ is the shear viscosity of the medium and ${\dot{\widetilde{\varepsilon }}}_{mS}$ is the shear strain rate on it caused by flow along the biointerface

The compressive stress caused by rearrangement of confluent epithelial monolayers corresponds to a few hundreds of Pa, while the compressive stress generated within multicellular spheroids corresponds to a few kPa (Notbohm et al. 2016; Kalli and Stylianopoulos 2018). The shear stress, caused by the movement of epithelial monolayers, corresponds to a few tens of Pa (Tambe et al. 2013). The shear stress generated along the epithelial-cancer biointerface has not yet been measured. If we postulate that it is a few tens of Pa, the corresponding dynamic friction coefficient would be ${C}_{F}\sim 0.01$, which corresponds to the transition between mixed and hydrodynamic lubrication regimes. In accordance with the fact that the epithelial-cancer interfacial tension contributes significantly to the epithelial friction, the dependence of the epithelial dynamic friction coefficient should be considered as a function of the Weber number.

## Conclusion

Friction plays an important role in human organs, both in their normal function and in the potential progression of disease. It is also important for the development of diagnostic and interventional medical devices. The presence of nanoscale surface roughness, viscoelastic or plastic deformations, wear, and lubrication all have an impact on single-cell functions. Frictional effects in soft matter systems have been quantified by a variety of frictional coefficients, such as (i) the dynamic friction coefficient, (ii) friction-skin drag, and (iii) pressure drag. These coefficients depend on the viscoelasticities of the two systems in contact and the relative velocity between them. Frictional effects along the biointerface between two solids have been discussed in the context of dynamic friction, because inertial stress can be neglected. However, the flow of fluid-like systems generates inertial stress, which has a feedback impact on frictional effects, and can be quantified in the form of drag coefficients. In the case of contact between viscoelastic materials, the situation becomes more complex because there is no clear boundary between solid-like and liquid-like behavior. Flow of viscoelastic fluids generates inertial stress, even at low Reynolds number. In this case, frictional effects along the biointerface between the viscoelastic liquid and the substrate matrix depend on two dimensionless numbers, i.e., the Reynolds number *R*_*e*_ and the Weissenberg number *W*_*i*_. The latter has been used as a measure of the system viscoelasticity. Low-${R}_{e}$ flows of viscoelastic fluids, such as polymer solutions, frequently satisfy the condition that $1\le {W}_{i}<10$. For low-${R}_{e}$ migration of epithelial monolayers on substrate matrices, however, ${W}_{i}\ll 1$. It is in accordance with the fact that the stress relaxation time corresponds to a time scale of minutes, whereas the strain change occurs on a time scale of hours. In this case, another dimensionless number could be more relevant for relating inertial to interfacial effects, such as the Weber number, ${W}_{e}$, which relates to the dilatational viscoelasticity.

In the context of frictional effects, several biological systems were considered: (i) epithelial tissues in contact with soft hydrogel-like implants; (ii) the collective migration of epithelial monolayers on substrate matrices; (iii) blood flow through blood vessels; and (iv) the movement of cancer cells past epithelial clusters, along with migration of epithelial cells within clusters. We conclude that.

- Frictional effects along the biointerface between epithelial tissue and a soft implant can be quantified by a dynamic frictional coefficient. This coefficient may vary across different lubrication regimes, ranging from boundary lubrication to hydrodynamic lubrication, as in the stated case of the contact lens in the ocular system. Fibrosis, induced by the immune response of the body to this friction, represents the cells’ way of protecting themselves against undesirable frictional shear stress. The immune response of soft tissue depends on the physical characteristics of implants such as the topography and rheological behavior of implants.
- Collective migration of epithelial monolayers on substrate matrices also generates frictional effects, which can be quantified by the dynamic frictional coefficient, depending on the matrix stiffness. Cells can self-regulate the friction by remodeling focal adhesions.
- Variations in the magnitude and direction of blood flow-induced shear stress can induce additional friction, quantified by the friction-skin drag coefficient. Cells are able to regulate the shear stress of blood to some extent. The shear stress can be increased by (i) cell movement against the blood flow and (ii) alterations in cell shape and adhesion contacts, leading to thinning of the cell monolayer and subsequent reduction in blood vessel diameter. The shear stress can be decreased by (i) cellular movement in the direction of blood flow and (ii) alterations in cell shape and adhesion contacts, leading to the thickening of the cell monolayer and subsequently an enlargement in the diameter of the blood vessel.
- Cancer cell movement past epithelial clusters (within co-cultured epithelial-cancer spheroids during the segregation process) induces frictional effects, which can be quantified by the pressure drag coefficient. Friction caused by the movement of epithelial cells within the cluster depends on the epithelial shear stress along the biointerface and the compressive stress accumulated within the cluster. Epithelial cells are able to protect themselves against frictional effects in two ways: (i) successive cell jamming-unjamming transitions, leading to energy dissipation accompanied by a decrease in shear stress, and (ii) establishment of a supracellular actin network along the cluster, which increase the resistance effects along the biointerface.

## Appendix

The linear constitutive model for describing the viscoelasticity of the cancer cell subpopulation in a confluent state is the Maxwell model expressed as (Pajic-Lijakovic 2021)

A1 $${{\widetilde{\sigma }}_{ci}}^{CCM}\left(r,{\tau }_{S},{\tau }_{L}\right)+{\tau }_{Rci} {{\dot{\widetilde{\sigma }}}_{ci}}^{CCM}\left(r,{\tau }_{S},{\tau }_{L}\right)={\upeta }_{ci}{\dot{\widetilde{\varepsilon }}}_{ci}\left(r,{\tau }_{L}\right)$$

where $i\equiv S,N$; $S$ is shear and $N$ implies normal; $r$ is the coordinate along the biointerface; ${\tau }_{S}$ is the short time of minutes; ${\tau }_{L}$ is the long-time of hours; ${{\widetilde{\sigma }}_{ci}}^{CCM}$ is the cancer mechanical stress (shear or normal) caused by collective cell migration; ${\widetilde{\varepsilon }}_{ci}$ is the cancer strain (shear or volumetric); ${\widetilde{\varepsilon }}_{cS}=\frac{1}{2}\left(\overrightarrow{\nabla }{\overrightarrow{u}}_{c}+\overrightarrow{\nabla }{{\overrightarrow{u}}_{c}}^{T}\right)$ is the cancer shear strain; ${\widetilde{\varepsilon }}_{cN}=(\overrightarrow{\nabla }\bullet {\overrightarrow{u}}_{c})\widetilde{I}$ is the cancer volumetric strain; ${\overrightarrow{u}}_{c}$ is the displacement field of the cancer subpopulation; $\widetilde{I}$ is the unity tensor; ${\dot{\widetilde{\varepsilon }}}_{ci}$ is the cancer strain rate; ${{\dot{\widetilde{\sigma }}}_{ci}}^{CCM}$ is the rate of cancer stress change; ${\upeta }_{ci}$ is the shear or bulk viscosity of the cancer subpopulation; and ${\tau }_{Rci}$ is the stress relaxation time of the cancer subpopulation.

This model for viscoelastic liquids describes stress relaxation from the initial value towards the cancer residual stress expressed as ${{\widetilde{\sigma }}_{cri}=\eta }_{ci}{\dot{\widetilde{\varepsilon }}}_{ci}$, under constant strain rate conditions ${\dot{\widetilde{\varepsilon }}}_{ci}$, such that the strain rate itself cannot relax. Stress relaxation occurs through multiple short-time cycles, each with a constant strain rate, while the change in strain rate happens over a longer time period. The key feature of the Maxwell model is its elastic behavior at short time scales (minutes) and viscous behavior at longer time scales (hours). This model considers both viscous and elastic properties in stress relaxation, with the residual stress being purely dissipative.

The linear constitutive model for describing the viscoelasticity of the epithelial subpopulation in a confluent state is the Zener model written in the form (Pajic-Lijakovic 2021):

A2 $${\widetilde{\sigma }}_{ei}\left(r,{\tau }_{S},{\tau }_{L}\right)+{\tau }_{Rei} {\dot{\widetilde{\sigma }}}_{ei}\left(r,{\tau }_{S},{\tau }_{L}\right)={E}_{ei}{\widetilde{\varepsilon }}_{ei}\left(r,{\tau }_{L}\right)+{\eta }_{ei}{\dot{\widetilde{\varepsilon }}}_{ei} \left(r,{\tau }_{L}\right)$$

where $i\equiv S,N$; $S$ is shear and $N$ is volumetric; ${\widetilde{\sigma }}_{ei}$ is the epithelial mechanical stress (shear or normal); ${\widetilde{\varepsilon }}_{ei}$ is the epithelial strain (shear or volumetric); ${\widetilde{\varepsilon }}_{eS}=\frac{1}{2}\left(\overrightarrow{\nabla }{\overrightarrow{u}}_{e}+\overrightarrow{\nabla }{{\overrightarrow{u}}_{e}}^{T}\right)$ is the epithelial shear strain; ${\widetilde{\varepsilon }}_{eN}=(\overrightarrow{\nabla }\bullet {\overrightarrow{u}}_{e})\widetilde{I}$ is the epithelial volumetric strain; ${\overrightarrow{u}}_{c}$ is the displacement field of the cancer subpopulation; $\widetilde{I}$ is the unity tensor; ${\dot{\widetilde{\varepsilon }}}_{ei}$ is the epithelial strain rate; ${\dot{\widetilde{\sigma }}}_{ei}$ is the rate of epithelial stress change; ${\upeta }_{ei}$ is shear or bulk viscosity of the epithelial subpopulation; ${E}_{ei}$ is the Young’s or shear modulus of the epithelial subpopulation; and ${\tau }_{Rei}$ is the stress relaxation time of the epithelial subpopulation.

This model for viscoelastic solids describes stress relaxation from an initial value towards the residual stress of ${\widetilde{\sigma }}_{rei}={E}_{ei} {\widetilde{\varepsilon }}_{ei}$ under constant strain ${\widetilde{\varepsilon }}_{ei}$ and strain relaxation under constant stress. This stress change occurs through multiple short-time cycles under constant strain, while strain change occurs over a longer time period. The ability of strain to relax is a fundamental characteristic of viscoelastic solids, and the Zener model is known for its viscous behavior on short time scales and elastic behavior on long time scales. In this model, stress relaxation encompasses both viscous and elastic behavior, with the residual stress being purely elastic.

## Abbreviations

${A}_{H}$: Projected surface normally to the direction of fluid flow
$${A}_{int}$$: Interfacial area
${C}_{D}^{skin}$: Friction-skin drag coefficient
${C}_{F}$: Dynamic frictional coefficient
${C}_{Fe}$: Dynamic frictional coefficient of epithelial tissues
${C}_{D}^{press}$: Pressure drag coefficient
${e}_{a}$: Adhesion energy between epithelial and cancer subpopulations
$${\overrightarrow{F}}_{F}$$: Frictional force
$${\overrightarrow{F}}_{D}$$: Drag force
$\overrightarrow{n}$: Unit normal vector
$$\Delta P$$: Contact pressure
$\overrightarrow{t}$: Unit tangential vector
${\overrightarrow{u}}_{c}$: Displacement of cancer subpopulation along the biointerface
${\overrightarrow{u}}_{e}$: Displacement of epithelial subpopulation along the biointerface
$\overrightarrow{v}$: Relative velocity between solids along the interface
${\overrightarrow{v}}_{c}$: Velocity of cancer cells
${\overrightarrow{v}}_{e}$: Velocity of epithelial cells
${\overrightarrow{v}}_{l}$: Velocity of fluid
${\overrightarrow{v}}_{p}$: Velocity of polymers
$${\overrightarrow{v}}_{slip}$$: Slip velocity
$\overrightarrow{z}$: Average deformation of surface asperities

## Greek symbols

${{\widetilde{\varepsilon }}_{surf}}^{s}$: Shear strain along the interface
${\dot{\widetilde{\varepsilon }}}_{lS}$: Fluid shear rate
${\widetilde{\varepsilon }}_{cN}$: Volumetric strain of cancer subpopulation
${\widetilde{\varepsilon }}_{eN}$: Volumetric strain of epithelial subpopulation
${\gamma }_{ce}$: Epithelial-cancer interfacial tension
${\gamma }_{e}$: Epithelial surface tension
${\gamma }_{c}$: Cancer surface tension
$\xi$: The Stokes friction coefficient
${{\widetilde{\sigma }}_{crN}}^{CCM}$: Normal residual stress of cancer cells caused by collective cell migration along the biointerface
${{\widetilde{\sigma }}_{erN}}^{CCM}$: Normal residual stress of epithelial cells caused by collective cell migration along the biointerface
${{\widetilde{\sigma }}_{erS}}^{CCM}$: Residual shear stress of epithelial cells caused by collective cell migration
${\widetilde{\sigma }}_{fr}$: Frictional shear stress
$${\widetilde{\sigma }}_{in}$$: Inertial stress
${{\widetilde{\sigma }}_{Sm}}^{vis}$: Shear stress caused by flow of liquid medium along the biointerface
${\widetilde{\sigma }}_{Sre}$: Viscoelastic residual shear stress of epithelial tissue
${\widetilde{\sigma }}_{surf}$: Total interfacial stress, which accounts for shear and normal contributions
${{\widetilde{\sigma }}_{surf S}}^{l}$: Fluid shear stress
${{\widetilde{\sigma }}_{surf}}^{p}$: Polymer contribution to the stress
${{\widetilde{\sigma }}_{surf N}}^{p}$: Normal contribution to the polymer stress
${{\widetilde{\sigma }}_{surf S}}^{p}$: Shear contribution to the polymer stress
${{\widetilde{\sigma }}_{surf}}^{s}$: Shear stress along the interface
${\widetilde{\sigma }}_{surf ss}$: Steady value of the interfacial shear stress
${{\widetilde{\sigma }}_{surf}}^{N}$: Normal stress on the interface
$\Delta {\widetilde{\sigma }}_{Nr}$: Normal residual stress difference between epithelial and cancer subpopulations along the biointerface
